# Bioimpedance Vector Patterns Changes in Response to Swimming Training: An Ecological Approach

**DOI:** 10.3390/ijerph17134851

**Published:** 2020-07-06

**Authors:** Joana F. Reis, Catarina N. Matias, Francesco Campa, José P. Morgado, Paulo Franco, Pedro Quaresma, Nuno Almeida, Dalia Curto, Stefania Toselli, Cristina P. Monteiro

**Affiliations:** 1 Faculdade de Motricidade Humana, Laboratory of Physiology and Biochemistry of Exercise, Universidade de Lisboa, 1499-002 Cruz Quebrada-Dafundo, Portugal; joanareis@fmh.ulisboa.pt (J.F.R.); cmatias@fmh.ulisboa.pt (C.N.M.); jmorgado@fmh.ulisboa.pt (J.P.M.); pedromquaresma@gmail.com (P.Q.); nrcalmeida@gmail.com (N.A.); dalia_serrasqueiro@outlook.com (D.C.); cmonteiro@fmh.ulisboa.pt (C.P.M.); 2 Interdisciplinary Center for the Study of Human Performance (CIPER), Faculdade de Motricidade Humana, Universidade de Lisboa, 1499-002 Cruz Quebrada-Dafundo, Portugal; 3 Department for Life Quality Studies, University of Bologna, 47921 Rimini, Italy; 4 Instituto Superior de Ciências Educativas, 1500-210 Lisbon, Portugal; 5 Universidade Europeia, 1500-210 Lisbon, Portugal; 6 Federação Portuguesa de Natação, 1500-210 Lisbon, Portugal; paulo.franco@fpnatacao.pt; 7 Department of Biomedical and Neuromotor Science, University of Bologna, 40125 Bologna, Italy; stefania.toselli@unibo.it

**Keywords:** BIVA, body composition, phase angle, R-Xc graph, vector length

## Abstract

Background and aim: Monitoring bioelectric phase angle (PhA) provides important information on the health and the condition of the athlete. Together with the vector length, PhA constitutes the bioimpedance vector analysis (BIVA) patterns, and their joint interpretation exceeds the limits of the evaluation of the PhA alone. The present investigation aimed to monitor changes in the BIVA patterns during a training macrocycle in swimmers, trying to ascertain if these parameters are sensitive to training load changes across a 13-week training period. Methods: Twelve national and international level swimmers (four females; eight males; 20.9 ± 1.9 years; with a competitive swimming background of 11.3 ± 1.8 years; undertaking 16–20 h of pool training and 4–5 h of dry-land training per week and 822.0 ± 59.0 International Swimming Federation (FINA) points) were evaluated for resistance (R) and reactance (Xc) using a single frequency phase sensitive bioimpedance device at the beginning of the macrocycle (M1), just before the beginning of the taper period (M2), and just before the main competition of the macrocycle (M3). At the three-time assessment points, swimmers also performed a 50 m all-out first stroke sprint with track start (T50 m) while time was recorded. Results: The results of the Hotelling T^2^ test showed a significant vector displacement due to simultaneous R and Xc changes (*p* < 0.001), where shifting from top to bottom along the major axis of the R-Xc graph from M1 to M2 was observed. From M2 to M3, a vector displacement up and left along the minor axis of the tolerance ellipses resulted in an increase in PhA (*p* < 0.01). The results suggest a gain in fluid with a decrease in cellular density from M1 to M2 due to decrements in R and Xc. Nevertheless, the reduced training load characterizing taper seemed to allow for an increase in PhA and, most importantly, an increase of Xc, thus demonstrating improved cellular health and physical condition, which was concomitant with a significant increase in the T50 m performance (*p* < 0.01). Conclusions: PhA, obtained by bioelectrical R and Xc, can be useful in monitoring the condition of swimmers preparing for competition. Monitoring BIVA patterns allows for an ecological approach to the swimmers’ health and condition assessment without resorting to equations to predict the related body composition variables.

## 1. Introduction

Endurance sports such as swimming typically require overload periods followed by a reduction of training load, or taper, designed to promote adaptations and obtain optimal performance in major competitions [1,2]. This process naturally encompasses training periods where fatigue affects performance whilst avoiding the counterproductive impact of non-functional overreaching and overtraining [3,4].

Given the complexity of athletic preparation and the required balance between fatigue and adaptation, several physical, physiological, and psychosocial measures are typically employed to help monitor and manage elite athletes during the different training phases of the season [5,6,7,8]. Another aspect that affects performance and can occur after a training macrocycle is an inadequate recovery of cellular homeostasis, as it can lead to fatigue of motor units and thus the potential recruitment of less efficient motor units in order to maintain performance and power output [9]. Moreover, a balance of fluids and hydration status play an important role, as hypohydration may affect physical function, cognitive performance, and health status [10,11]. However, the taper phase, occurring in the weeks before the major competition of the macrocycle, allows for physiological and psychological recovery from the accumulated training stress through a marked decrease of the training load [12]. Literature has shown that, at the neuromuscular level, taper promotes an increase in muscular strength and power as well as muscle fiber size adaptations [13,14,15]. Additionally, taper has been shown to allow for supercompensation of muscle glycogen storage, which can affect the intracellular water volume in muscle cells and ultimately performance [12].

The analysis of body composition is fundamental in sports because of its relevance to athletes’ health and performance [16,17]. Recently, attention has been given to measuring phase angle (PhA)—a non-invasive, simple measurement using bioelectrical impedance analysis (BIA)—which allows for an evaluation of body composition at the whole-body level [18]. Indeed, PhA is considered a valuable indicator of cellular health and, as it is derived purely from electrical properties of the tissue, it avoids typical concerns associated with BIA using prediction equations. PhA is calculated from the arctangent of the ratio between the resistance (R) and the reactance (Xc) from BIA, where R arises from extracellular water (ECW) and intracellular water (ICW), while Xc arises from cell membranes. Therefore, Xc is the cell membrane’s capacity for capturing an electric load and releasing it in a second moment after a brief delay; it could be compared to a vessel-capacitance-like property. However, the analysis of PhA by itself can lead to interpretation errors. In fact, groups of individuals characterized by quite identical PhA, which represent the ICW/ECW ratio, may show a different body structure [19]. The bioimpedance vector analysis (BIVA), which considers both R and Xc values on the R-Xc graph [20], appears to be more accurate, as it considers both influential variables, PhA and vector length definite, as the BIVA patterns [19]. The use of BIVA in the sports field has grown in recent years [21,22] as its reliability in monitoring the change in fluids during a competitive season [23] as well as in the short term [10,24] has been recognized. In particular, Carrasco-Marginet [25] suggested how BIVA, being a practical and fast method, can be used to monitor hydration status after a swimming competition. However, to the best of our knowledge, no studies have monitored changes in BIVA patterns during a preparation cycle in swimmers. Specifically, there is no longitudinal study that addresses the effect of traditional swimming training in these parameters. It would be particularly helpful for coaches and physiologists to determine if BIVA analysis can non-invasively discriminate the fatigue state of swimmers across the build-up and taper phases before a major competition.

Therefore, our aim was to monitor BIVA patterns during a training macrocycle in swimmers, trying to ascertain if these parameters are sensible to training load changes across a 13-week training period designed to promote peak performance. We also aimed to determine if BIVA patterns change in parallel with swimming performance in a sprint time-trial. Our hypothesis was that vector changes could occur during the different training phases considered in this study, leading to a shift in the axis corresponding to variations in the body composition and/or in the hydration status, therefore providing useful information for monitoring body composition, physical condition, and performance of swimmers.

## 2. Materials and Methods

### 2.1. Participants

An a priori power analysis was conducted to determine the sample size for the study, using G*Power software 3.1.9.2. Significance level and power were, respectively, set at 0.05 and 0.90, whereas expected effect size was assumed to be at least medium for PhA and T50m changes. The estimated sample size was thus 9 swimmers, but 3 additional subjects were involved to ensure availability of data in case of problems with data collection. Swimmers were recruited from the national training center and from one of the national top teams. Inclusion criteria were: above 18 years in age, training and competitive experience above 8 years, and having represented the national team at least once in the two years before the study. Twelve national and international level swimmers (4 females, 8 males), mean age 20.9 ± 1.9 years, with a competitive swimming background of 11.3 ± 1.8 years, undertaking 16–20 h of pool training and 4–5 h of dry-land training per week and 822.0 ± 59.0 International Swimming Federation (FINA) points in their best event were evaluated in this study. After receiving detailed information about the aim of the study and the possible risks of the investigation, the participants provided their written informed consent to participate. All procedures were approved by the Ethics Committee of the Faculty of Human Kinetics of the University of Lisbon (approval code: 039/15) and were conducted in accordance with the Declaration of Helsinki for human studies [26].

### 2.2. Study Design

This study used an observational design with a follow-up over a swimming training macrocycle lasting 13 weeks (between January and April). Swimmers followed the training program set by their technical team.

The evaluation of the swimmers was made at rest at the beginning of the second macrocycle (M1) of the season, immediately before the beginning of the taper period (M2), which occurred in the middle of week 10, and immediately before the main competition of the macrocycle at the end of week 13 (M3). Before M1, swimmers endured a two-week transition phase after the main competition of the first macrocycle of the year, where the swimmers did not train. At each moment of evaluation, body composition measurements were performed in the morning (07:00) after an overnight fast with no alcohol or stimulant beverage consumption or exercise participation taking place within the last 12 h. A performance test followed the body composition measurements after a standardize pre-exercise food intake to avoid extending the duration of the fasted state.

### 2.3. Body Composition Measurements

Weight and height were measured in the fasted state wearing a bathing suit without shoes to the nearest 0.1 kg and 0.1 cm (Secca, Hamburg, Germany).

The impedance measurements were performed by a phase-sensitive single-frequency bioimpedance analyzer (BIA 101 Anniversary, Akern, Florence, Italy), which applies an alternating current of 400 microA at 50 kHz. Before the BIA measurement, each subject rested in a supine position for ten minutes to stabilize body fluids. Afterwards, the measurements were made with the subjects in the supine position with a leg opening of approximately 45° compared to the median line of the body and the upper limbs positioned about 30° away from the trunk [19]. After cleaning the skin with alcohol, two Ag/AgCl low-impedance electrodes (Biatrodes, Akern Srl, Florence, Italy) were placed on the back of the right hand and two electrodes on the corresponding foot, with a distance of 5 cm between each other [19]. Vector length (VL) was calculated as (R^2^ + Xc^2^)^0.5^ and PhA as the arctangent of Xc/R x 180/π. BIVA was carried out using the classic methods, e.g., normalizing VL, R, and Xc for height (H) in meters. The R-Xc z score graph was used to plot the BIVA data of the swimmers separated by gender; in this approach, the mean bioimpedance vectors are considered in relation to their specific reference population [27]

Total body water (TBW), ECW, ICW, as well as fat-free mass (FFM) and fat mass (FM) were calculated using specific equation for athletes [28,29].

### 2.4. Performance Measurements

Performance evaluation was performed in an Olympic sized swimming pool. After standardized warm up, the swimmers performed a 50 m all-out best stroke sprint with track start (T50 m) and time was recorded with a stopwatch (Seiko S141, Tokyo, Japan) by two experienced timekeepers. The average of the measurements was considered.

### 2.5. Quantification of the Training Load

The training load of each session was assessed as previously described [30]. Briefly, the training load of each session was assessed by quantifying the volume (total amount of meters swum), the weighted volume (sum of the meters swum in each zone of intensity, multiplied by the respective index), and the arbitrary units of load (AUL) adapted from previous investigations [2,31,32]. The weekly load was characterized by the sum of the load of all the training sessions of each week.

### 2.6. Statistical Analysis

Descriptive statistics were applied to characterize the sample. All variables were checked for normality using Shapiro–Wilk test. All the resulting variables were normally distributed, with the exception of T50 m. General linear model was applied to check the potential interaction between sex and variables of interest. A repeated measures ANOVA (or Friedman when normality was not observed) was performed to compare body composition and performance parameters between the 3 evaluation time points. When a significant effect was detected at a significance level of *p* < 0.01, the Bonferroni test was used for post-hoc comparisons. The paired, one-sample Hotelling T^2^-test was performed to determine if the changes in the mean group vectors (measured at the first, the second, and the third time points) were significantly different from zero (null vector); a 95% confidence ellipse excluding the null vector indicated a significant vector displacement. All statistical calculations were computed using the Statistical Package for the Social Sciences (SPSS) version 25.0 (IBM Corp., Armonk, NY, USA). Significance was set at *p* < 0.05.

## 3. Results

No influence of sex was verified on the variables of interest; therefore, the sample was gathered as a whole for the statistical analysis for body composition and performance differences between moments of evaluation.

Compared to the swimmer’s personal best time in the 50 m, the T50 m represented 94.7 ± 4.8%; 96.0 ± 2.5% and 98.3 ± 2.0 in M1, M2, and M3, respectively.

From baseline (M1) to the end of the macrocycle (M3), FM% and T50m decreased, while PhA mean values increased (*p* < 0.01) in all athletes (Table 1). Furthermore, between M2 and M3, there was an upsurge in PhA concomitant with a decline in T50 m, representing an increase of performance of 3.36 ± 2.88%.

**Figure 1:**
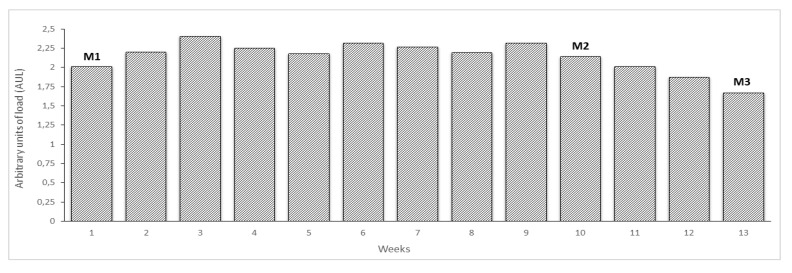
Training load (UAL) and evaluation moments during the 13 weeks macrocycle studied.

The training loads across the whole macrocycle and the time points of the evaluations are presented in Figure 1.

The beginning of the macrocycle was characterized by an increasing training load until week 3, followed by a maintenance period until week 10, where the M2 evaluation took place. Afterwards, the training load was progressively reduced preparing for the main competition of the macrocycle (M3). The average weekly volume between M1 and M2 was 71222 ± 8559 m and between M2 and M3 was 38848 ± 21461 m.

**Figure 2:**
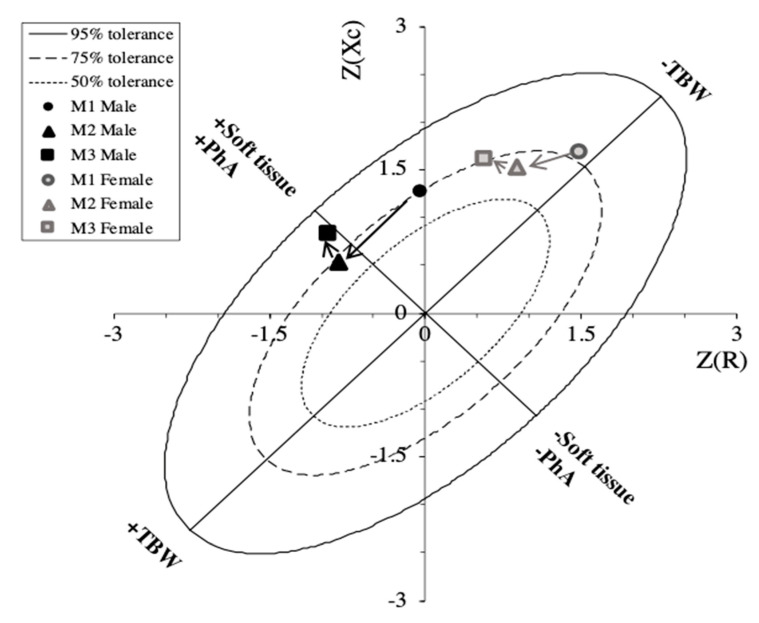
The bioimpedance vector analysis (BIVA) R-Xc z-score graph: Bioimpedance data are plotted on the RXc z-score graph after transformation of the impedance measurements from the athletes into bivariate z-scores (with respect to their reference population).

Figure 2 shows a R-Xc z-score graph with mean impedance vectors displacements between the three time points for the two sexes.

**Figure 3:**
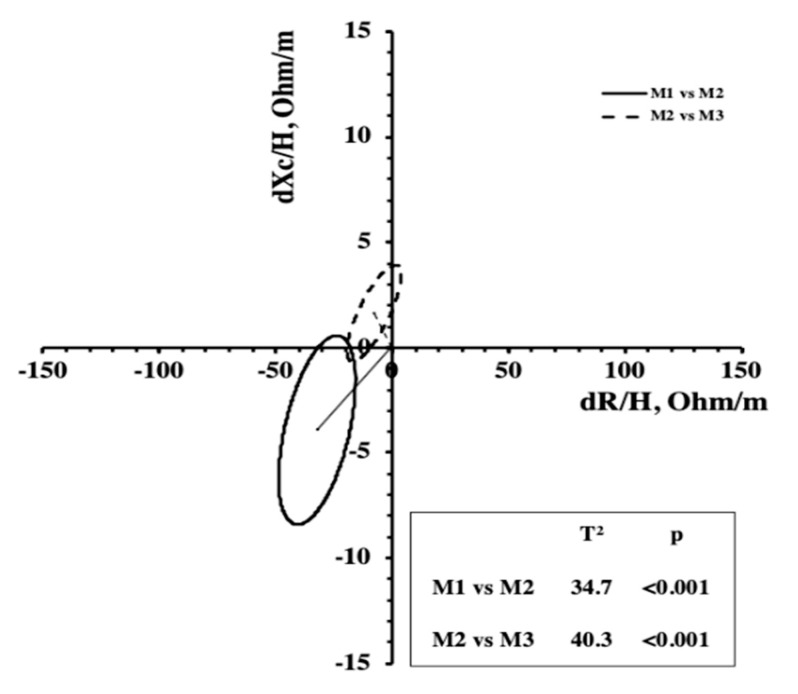
Mean displacements vector and results of the Hotelling’s T^2^ test.

Since no significant interaction (*p* > 0.05) between sex and time was found, the athletes were considered as a single group for the Hotelling’s T^2^ test, which takes into consideration the combined changes of R/H and Xc/H. Significant changes in BIVA patterns were identified from M1 to M2 due to reductions in R/H and Xc/H and from M2 to M3 (Figure 3).

## 4. Discussion

This study adds new knowledge to the relevance and the utility of BIVA in the field context along a swimming training season regarding its benefits in monitoring body composition, cellular integrity, and, therefore—although indirectly—performance. The main finding of the present investigation is that changes in BIVA patterns throughout a training macrocycle are associated with changes in body composition and performance in swimmers.

The R-Xc graph indicates a shift of the mean vector from top to bottom along the major axis of the tolerance ellipses from M1 to M2. Our results showed a simultaneous reduction of R/H and Xc/H, indicating a gain in fluid where a decrease in cellular density in comparison to M1 can be justified. However, this condition could also be due to the fatigue accumulated during the training phase. In fact, a significant shortening of the vector was found in previous studies along three weeks of a multistage road bicycle race, indicating fluid gain during the competition [33]. The authors attributed these results to muscle edema, hemodilution, release of water associated with muscle glycogen oxidation, and excess fluid intake [33]. At the final assessment point (M3), we noted a vector shift to the left along the minor axis of the tolerance ellipses on the R-Xc graph, resulting in an increase in PhA. Since the ICW/ECW ratio is positively related to PhA [23,34], the increase in the later could be due to the hypertrophy of the muscle fibers obtained at the end of the macrocycle before the competition due to the decreased training load and consequently enhanced recovery promoted in the taper period. Furthermore, as suggested by Castizo-Olier and colleagues [22], the vector shift to the left indicates increased body cell mass and fluid content. If we consider the BIVA changes from M2 to M3 (Figure 3), referring to an interval where the training load decreased, we still see a small reduction in R/H, suggesting an increase in soft tissue caused by the supercompensation resulting from training. More importantly, an increase of Xc/H suggests improved cellular density, probably due to an increase in the size and the number of muscle cells (hypertrophy and hyperplasia, respectively) or possibly due to plasma volume expansion and enhanced glycogen storage, as suggested by Mascherini et al. [35], who monitored vector changes during a season in soccer players. This is in accordance with previous studies reporting the aforementioned physiological adaptations that also occurred following taper in endurance sports such as cycling, running, and swimming [14,36,37]. As highlighted in the study by Macherini et al. [35], with PhA being positively correlated with the ICW/ECW ratio, it can decrease following cell damage caused by an overreaching period and can increase after cell hypertrophy. In this study, PhA decreased during the preparatory period and then showed an increase before the competitive start to the soccer season.

Interestingly, in our study, PhA increased from M2 to M3 after the taper period as well as performance. Previous studies reported that lower PhA appears to be consistent with either cell death or an increase in the fragility of the cell’s membranes, whereas higher PhA is associated with large quantities of intact cell membranes and body cell mass [22,38]. Therefore, higher PhA values reflect a cell’s membrane integrity and better cell function [39]. It has been suggested that a lower PhA emerged as a useful predictor for impaired muscle function and muscle fatigue [40,41], and, conversely, it has been positively associated with improved power output in elite road cyclists [33]. In adult non-athletes, healthy or diseased, the bioelectrical vector shift to the left and PhA increase have also been associated with increases in strength and decreases in fat mass [39,42,43]. In this regard, BIVA data should be evaluated as a routine assessment, specifically PhA and vector displacement, as they may be useful for assessing function and body fluids in competitive athletes, and their integrated evaluation might contribute to identifying changes in body composition and gauging transient fatigue and reduced performance in athletes.

Despite the encouraging results, some limitations are present and should be considered, mostly emerging from the fact that this is an observational study that monitors elite athletes within their regular training; therefore, applying non-invasive and time efficient measurements could help coaches to program and control the training process. However, we acknowledge that further studies that describe more invasive physiological parameters that can monitor the underlying parameters of muscle fatigue or recovery status are necessary to investigate this topic. Additionally, trying not to compromise the training process or promote additional fatigue, especially in the evaluation preceding the main competition of the macrocycle, we evaluated performance as the time in a 50 m sprint, which could have underestimated the performance improvements because the swimmers were short and middle-distance specialists. One should also consider that some female swimmers were not in the same menstrual phase in all the evaluation moments, which can influence body fluids retention and introduce some variability in the data obtained. Furthermore, future research should include more evaluation time points within the taper, which could help establish the suitable duration of this training phase and ultimately increase the performance improvement.

Bearing in mind these limitations, the results of the present investigation show that the combined changes of PhA and the vector length representing the BIVA patterns can be useful in monitoring the condition of the athlete preparing for the competition. In particular, decreases and increases in PhA may indicate fatigue situations and subsequent recovery following supercompensation after the training period, respectively. In this regard, further studies that consider physiological parameters that can monitor the underlying parameters of muscle fatigue and that introduce changes in the recovery period are necessary to investigate this topic further.

## 5. Conclusions

Using an ecological approach, this study showed that the interpretation of PhA and vector displacements within the R-Xc graph can be a practical way of determining the evolution of elite swimmers among the different training phases during a season, as it can monitor both body composition and performance of the athletes. In particular, a lower-left vector displacement without changes in PhA was observed during the build-up phase. Subsequently, due to the recovery and the modulation of the training load, the adaptations sought through training were reflected in an upper-left vector displacement and therefore an increase in PhA. This occurred in combination with an improvement in performance.

## Figures and Tables

**Table 1:** Changes in body composition, bioelectric and performance variables, from the beginning of the season, to before the beginning of the taper period (M2), and to just before the main competition of the macrocycle (M3) in the swimmers.

Variable	M1	M2	M3	ANOVA/Friedman	
				F/X^2^	*p*
Weight (kg)	68.6 ± 9.7	67.8 ± 9.6	67.7 ± 9.3	0.0	0.96
Height (m)	1.77 ± 0.1	-	-	-	-
Fat mass (kg)	16.3 ± 2.2	14.7 ± 2.0	14.2 ± 2.5	2.8	0.72
Fat mass (%)	23.9 ± 2.1	21.5 ± 2.5	20.8 ± 2.8 *	4.0	0.01
FFM (kg)	52.3 ± 8.1	53.9 ± 8.5	53.5 ± 8.2	0.2	0.80
TBW (L)	37.8 ± 5.3	38.6 ± 5.7	38.9 ± 5.6	0.1	0.86
ECW (L)	16.1 ± 2.0	16.5 ± 2.2	16.5 ± 2.0	0.1	0.84
ICW (L)	21.6 ± 3.4	22.1 ± 3.5	22.4 ± 3.5	0.1	0.85
R/H (Ohm/m)	319.6 ± 45.9	287.4 ± 39.7	279.6 ± 37.7	3.2	0.05
Xc/H (Ohm/m)	72.5 ± 11.8	67.7 ± 6.3	68.3 ± 6.1	1.3	0.27
VL/H (Ohm/m)	322.1 ± 46.0	290.1 ± 39.8	289.8 ± 39.9	2.3	0.11
PhA (º)	7.2 ± 0.6	7.3 ± 0.6	7.9 ± 0.7 *^,#^	4.7	0.01
T50 m (s)	28.1 ± 4.2	27.9 ± 3.7	27.1 ± 3.4 *^,#^	30.9	<0.01

Note: Data are reported as mean ± standard deviation. FFM = fat-free mass; TBW = total body water; ECW = extracellular water; ICW = intracellular water; R/H = resistance standardized for height, Xc/H = reactance standardized for height, VL/H = vector length standardized for height, PhA = phase angle; T50m-50 m all-out first stroke sprint with track start. * significantly different from M1; ^#^ significantly different from M2; *p* < 0.01.
